# UNVEILING THE COMPLEX REALITIES: UNDERSTANDING SLEEP AND FATIGUE CHARACTERISTICS OF END-OF-LIFE CAREGIVERS

**DOI:** 10.1093/geroni/igad104.3684

**Published:** 2023-12-21

**Authors:** Hyeyoung Park, Sunghoon Lee, Amelia Bailey, Yunda Liu, Cynthia Jacelon

**Affiliations:** University of Massachusetts Amherst College of Nursing, Amherst, Massachusetts, United States; University of Massachusetts Amherst, Amherst, Massachusetts, United States; Brown University, Providence, Rhode Island, United States; University of Massachusetts Amherst, Amherst, Massachusetts, United States; University of Massachusetts Amherst College of Nursing, Amherst, Massachusetts, United States

## Abstract

This mixed methods multi-case study explores sleep and fatigue characteristics among end-of-life caregivers. The research sample encompasses cases aiding individuals with advanced (n=4) and chronic illnesses (n=1), including various caregiver demographics such as gender, age groups (19-64 and 65+), and health conditions. Data collection involves comprehensive surveys capturing demographics, care recipient symptom intensity, caregiver depressive symptoms, caregiving stress, caregiver sleep quality, and caregiver fatigue level. The study integrates data from a 30-day Personal Sleep Monitoring Device (PSMD) and a 14-day sleep journal, enriched by insights from semi-structured interviews and field notes. The outcomes indicate a PSQI (Pittsburgh Sleep Quality Index) score of 6.1, accompanied by 7.44 hours of sleep recorded by the PSMD. There were approximately 20.5 nocturnal awakenings within 30 days, with average fatigue at 55.28 and daily fatigue at 49.66. Objective sleep outcomes were consistent among caregivers, while their own sleep ratings ranged from “Fairly Bad” to “Very Good.” Themes for each case were formulated to delve into the context of these sleep and fatigue characteristics. These themes encompass adaptive leadership, linchpin roles, emotional labor management, self-reliance, and vigilance. A prevailing overarching theme is the perception of “Not having control.” A significant finding underscores the dynamic influence of care recipients’ daily statuses on caregivers’ sleep quality and fatigue levels, leading to unpredictable outcomes. This study offers profound insights into end-of-life caregivers’ intricate journeys, highlighting individual differences and the dynamic nature of caregiving roles, while recognizing the importance of tailored interventions for enhancing caregiver’s well-being and caregiving effectiveness.

